# Anterolateral corner of knee: Current concepts

**DOI:** 10.1002/jeo2.70172

**Published:** 2025-02-10

**Authors:** Amit Meena, Manish Attri, Luca Farinelli, Vicente Campos, Karan Rajpal, Riccardo D'Ambrosi, Shahbaz Malik, Darren de Sa, Christian Fink, Sachin Tapasvi

**Affiliations:** ^1^ Department of Orthopedics Shalby Hospital Jaipur India; ^2^ Department of Orthopaedics Santosh Medical College and Hospital Ghaziabad India; ^3^ Department of Clinical and Molecular Sciences, Clinical Orthopedics Università Politecnica delle Marche Ancona Italy; ^4^ IRCCS INRCA Ancona Italy; ^5^ Department of Orthopedics Hospital Curry Cabral Lisboa Portugal; ^6^ The Orthopaedic Speciality Clinic Pune India; ^7^ IRCCS Istituto Ortopedico Galeazzi Milan Italy; ^8^ Dipartimento di Scienze Biomediche per la Salute Università degli Studi di Milano Milan Italy; ^9^ Worcestershire Acute Hospitals NHS Trust Worcester UK; ^10^ Division of Orthopaedic Surgery, Department of Surgery McMaster University Medical Centre Hamilton Ontario Canada; ^11^ Gelenkpunkt – Sports and Joint Surgery, FIFA Medical Centre of Excellence Innsbruck Austria

**Keywords:** anterolateral capsule, anterolateral instability, anterolateral ligament, knee, rotatory instability, rotatory laxity

## Abstract

The anatomy of the antero‐lateral corner (ALC) has been the topic of recent interest, as evidenced by the increasing number of publications. Knowledge needs to be improved amongst clinicians regarding the anatomy and biomechanical function of this vital structure and its implications on the rotational stability of the knee. There has yet to be a consensus on the role of surgical procedures and their indications for addressing the instability associated with the injury to these structures. Through this article, the authors have tried to outline the existing literature regarding Anterolateral knee instability, the associated structures, and the management of its injuries, emphasising the role of the anterolateral capsule and reconstructive procedures in combined ligamentous knee injuries.

## Level of evidence

Level of Evidence V.

**Abbreviations**
ACL: anterior cruciate ligament
ACLR: anterior cruciate ligament reconstruction
ALC: anterolateral complex
ALL: anterolateral ligament
ALLR: anterolateral ligament reconstruction
ALRI: anterolateral rotational instability
FCL: fibular collateral ligament
ITB: iliotibial band
LCL: lateral collateral ligament
LEAP: lateral extra‐articular procedure
LET: lateral extra‐articular tenodesis
MRI: magnetic resonance imaging
OA: osteoarthritis
PKF: proximal Kaplan fibres

## INTRODUCTION

There is an increase in the incidence of anterior cruciate ligament (ACL) reconstruction surgeries, especially among young athletes and females [76]. Graft failure occurs in up to 20% of patients undergoing ACL reconstruction surgery despite the advancements in surgical techniques and our enhanced understanding of ACL anatomy [57]. The main cause for ACL failure can be attributed to technical failures, biology, and trauma [95]. Injury to ACL and other extra‐articular structures may lead to rotatory knee instability which has been recognised as a cause of graft failure in up to 25% of patients undergoing ACLR [12, 91]. These extra‐articular structures of the anterolateral aspect of the knee are referred to as the anterolateral complex (ALC). This persistent knee rotatory instability can be addressed by augmentation procedures as lateral extra‐articular tenodesis (LET).

Thorough knowledge of the anatomy of the ALC, its biomechanics and outcomes of ACL reconstruction with lateral‐based augmentation is important to understand the role of ALC and the patient groups who can benefit from lateral augmentation with concomitant ACL reconstruction.

## THE ENIGMATIC ALL: DEFINITION AND FUNCTION

The awareness regarding the anterolateral corner (ALC) has increased since the identification of anterolateral ligament (ALL) [16]. The consensus about ALL's function and its existence are lacking. Whereas some authors completely deny its existence [84, 96], others have identified this ligament [14, 42]. The difference in dissection techniques may be the reason for this disagreement.

It is to be noted that ACL tears associated with ALL injuries have increased knee rotational instability, causing increased internal rotation of the tibia resulting in poor functional outcomes after isolated ACL reconstruction (ACLR) [28, 29]. These isolated ACLRs are associated with increased meniscus tears and subsequent development of osteoarthritis [92]. Several anatomical ACLR techniques have been described to improve the residual rotational instability by creating a more oblique femoral tunnel and double‐bundle ACLR. Still, no significant rotationally stabilising effect has been noted [31].

## A BRIEF HISTORY OF ALL: FROM DISCOVERY TO CURRENT UNDERSTANDING

The ALL was first described by Segond in 1879 in a report of an avulsion fracture of the tibia [102]. Hughston et al. rediscovered ALL in the 1970s as a structure called “mid‐third lateral capsular ligament” that was connected to the lateral meniscus and commented upon its role in rotational instability around the knee [49]. An anterior oblique band of the fibular collateral ligament (FCL) was also described in 1970 [53], In contrast, Terry et al. [93] claimed that capsulo‐osseous and superficial layers of the iliotibial band (ITB) were the main structures determining the rotational stability of the lateral side of the knee. These capsulo‐osseous layers were described to act as ALLs in 2007 [97]. Claes et al. [16] gave an in‐depth insight into ALL, describing its anatomical structures and their function.

## ALL ANATOMY: STRUCTURE AND ATTACHMENTS

**Figure 1:**
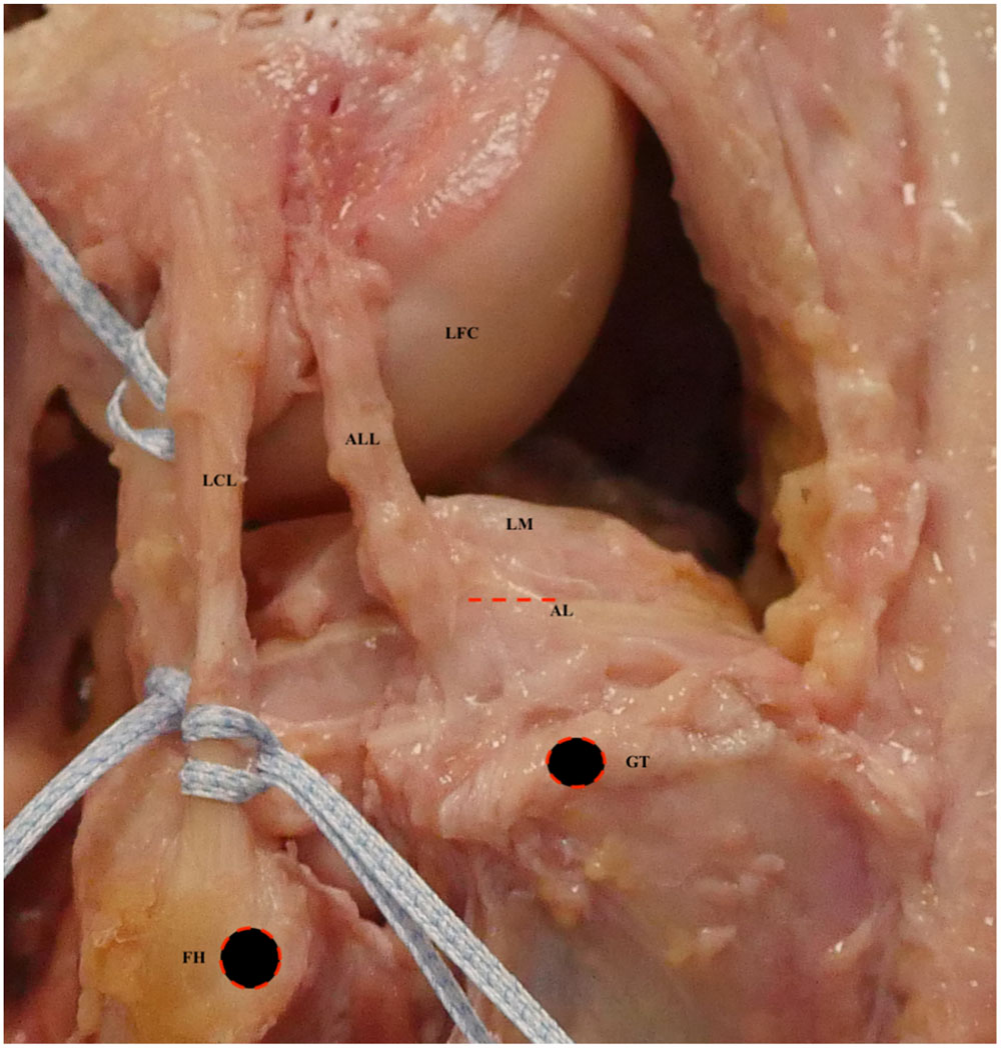
Anatomy of the anterolateral ligament (ALL). It originates in the lateral epicondyle, anterior and distal to the lateral collateral ligament (LCL) origin. It follows an anteroinferior course toward the tibia, with tibial insertion between the Gerdy tubercle (GT) and the fibular head (FH), slightly more than 5 mm below the articular cartilage of the lateral tibial plateau. AL, articular line; LFC, lateral femoral condyle; LM, lateral meniscus.

**Figure 2:**
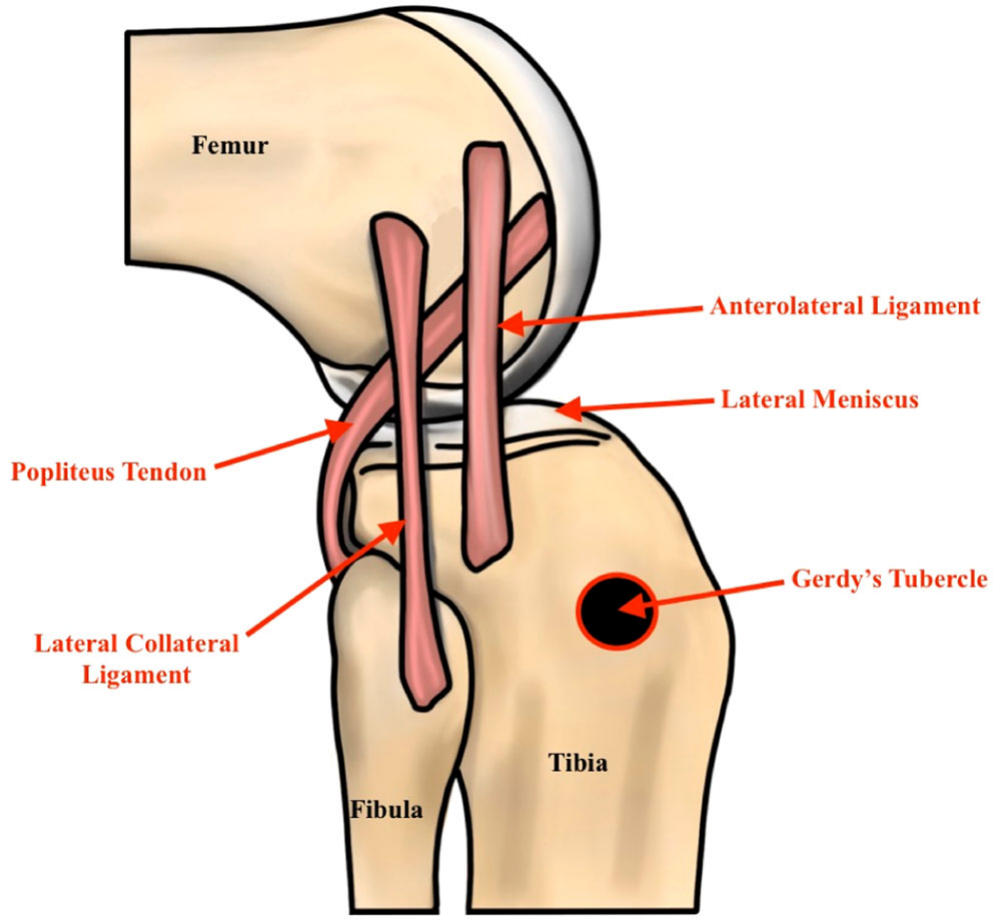
The anterolateral ligament (ALL) has close interaction with the popliteus tendon and the iliotibial band.

The ALL has been described as an extracapsular broad, translucent fibrous band running from the prominence of lateral femoral epicondyle between FCL insertion posteriorly and insertion of popliteus tendon anteriorly to the area between Gerdy's tubercle anteriorly and fibula the posteriorly (Figures 1 and 2) [5, 17, 75]. It has connections to the lateral intermuscular septum and FCL via superficial and posterior fibres with fibres that connect the ligament to the lateral meniscus [16]. It can be compared to the deep medial collateral ligament (dMCL). The position of the ALL is not fixed, especially its femoral insertion [87]. The cause for this discrepancy can be attributed to different dissection techniques in different studies or individual differences between different specimens.

A study describes the ALL as made up of two separate bundles: the superficial bundle inserts on the area proximal and posterior to the femoral epicondyle, while the deep bundle inserts on the centre of the femoral epicondyle [44]. It also describes its close association with the ITB. It demonstrates that the ALL works along with an ACL to limit the internal rotation of the tibia.

The Kaplan fibres are described as a deep, posterior portion of the ITB having attachment to the femoral metaphysis, in close proximity to the branches of the superior genicular artery, it is divided into two bundles, the proximal Kaplan fibres (PKF) and the distal Kaplan fibres (DKF). These along with the ITB act as secondary passive stabilisers of the knee [2]. Their injuries can be identified on MRI and the rate of the concomitant Kaplan fibres injury in ACL tear has been reported to be 17.6%–60% [29, 83].

The capsule‐osseous layer has been described as an arcuate fibre tract extending from the intermuscular septum over the lateral supracondylar region of the femur to Gerdy's tubercle, forming a sling around the posterolateral aspect of the distal femur. It is shown to have a complementary role in limiting internal rotation but is distinct from ALL [39].

## HISTOLOGY

Microscopically, the collagen pattern of ALC is organised into bundles, implying it to be a combination of multiple lateral joint capsule thickening, unlike the homogenously arranged bundles of an ACL [10], However, a more consistent collagenous pattern can be noted in the femoral and tibial attachments. The transition between mineralised cartilage and ALC shows ligamentous tissue having peripheral nervous innervation and mechanoreceptors [10].

Another study showed dense connective tissue with a fibrous arrangement and scanty cellular material; [42] however, its structural organisation is irregular compared to the FCL [22].

## ALL IMAGING: RADIOLOGICAL FINDINGS AND TECHNIQUES

Various radiological studies have described the origin and insertion of a potential ligamentous structure in the ALC of the knee [43, 81]. For instance, radiographs can point towards Segond fracture, which is thought to be due to avulsion resulting from attachment of ligamentous structure on the lateral aspect of proximal tibia [43]. This may suggest the involvement of the ITB insertion [18].

An MRI is a gold standard investigation for visualising the ALL, although the structures can also be well appreciated on USG [15]. In a study, a 2–4‐mm thick structure was appreciated in the central third of the latent capsule in 30% of the specimens. This ligamentous structure has been visualised in as high as 97.4% and 100% of the cases in other studies [45].

ALL tears have been found in up to 40% of patients with complete ACL tears, whereas in patients with partial ACL tears, ALL tears have not been visualised on imaging [59]. These MRI findings can be correlated with clinical examination and tests for rotational instability [48], demonstrating the role of these structures in providing anterolateral stability to the knee.

## ALL BIOMECHANICS: UNDERSTANDING ITS ROLE IN KNEE STABILITY

Lateral instability of the knee, although it occurs less frequently, is more disabling as compared to the medial side [6]. The ALC is believed to be the secondary stabiliser for internal rotation and anterior translation of the lateral compartment of the knee [68]. The ALL limits the internal rotation when the knee is flexed beyond 35°, whereas it provides only minimal resistance to anteroposterior translation [74]. Certain researchers have shown that the ALL acts as a stabilising structure in internal rotation and knee extension [79].

The roll glide mechanism of the lateral epicondyle does not allow us to find isometric femorotibial connections [85]. Hence, the ligamentous structures of the knee are not believed to possess isometric behaviour, leading to the failure of various surgical techniques that aim at isometric reconstruction. Generally, the structures posterior to the femoral epicondyle are tight in extension. In contrast, there is increased strain on the structures anterior to the femoral epicondyle when flexing the knee.

**Figure 3:**
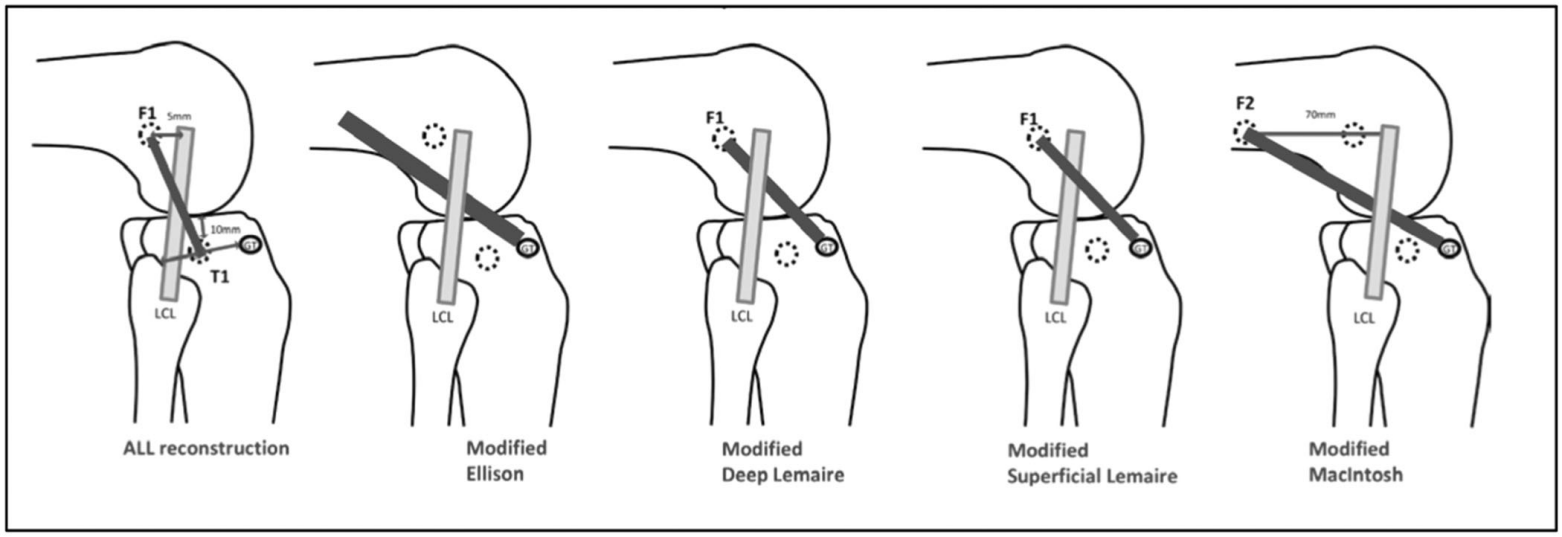
Various anterolateral extra‐articular procedures. F1: Femoral tunnel position for ALL reconstruction and Lemaire procedures (5 mm posterior and proximal to the femoral epicondyle). F2: Femoral tunnel position for modified MacIntosh procedure (70 mm posterior and proximal to the femoral epicondyle). T1: Tibial tunnel position for ALL reconstruction (equidistant from the centre of Gerdy tubercle (GT) and the anterior margin of the fibular head and 10 mm distal to the joint line). ALL, anterolateral ligament; LCL, lateral collateral ligament.

In a study, after sectioning ALL, it was found that at 20° of knee flexion, there was no increase in tibial internal rotation. Yet, at 90° of knee flexion, the internal rotation was increased, showing the role of the ITB in rotational control of the knee at lower degrees of knee flexion [89]. Similarly, other authors [24, 73] confirmed that ALL limits internal rotation at higher degrees of knee flexion and has no role [34] when the knee is flexed up to 20°–30°. Hence, it would not be wrong to suggest that different structures of ALC are responsible for preventing antero‐lateral rotational instability (ALRI) at varying angles of knee flexion (Figure 3).

It is also important to note that the inference about ALL function from these in vitro studies may differ from its in vivo function because of specific connections between ALL and the ITB by means of Kaplan fibres, ITB might influence the ALL tension dynamically in vivo [24]. This leads to an increase in the length of ALL fibres with increased knee flexion as demonstrated by a cadaveric study [101]. Multiple studies have shown the role of Kaplan fibres, which connect the ITB to ALC, acting as internal rotation stabilising ligaments. If these are severed, the ITB loses its function and ability to control the internal rotation of the tibia [36, 56, 61].

To summarise, in ACL deficient knees, ALL controls internal rotation at higher knee flexion angles. In contrast, deep fibres of an ITB through Kaplan fibres control internal rotation near the extension.

## NEED FOR LATERAL EXTRAARTICULAR PROCEDURES (LEAP)?

The anterior lateral ligament stabilises the knee against excessive pivot shift and internal rotation in an ACL‐deficient knee [60, 79]. But ALL is not the only structure which provides rotational stability. The Kaplan fibres consisting of two bundles also stabilise the knee against excessive internal rotation. In a study, the ACL, ALL and Kaplan fibres were sequentially sectioned to ascertain their function in ACL‐deficient knee. It was seen that ALL and/or Kaplan fibre sectioning resulted in a significant increase in anterior tibial translation, internal rotation and pivot shift. At 60°–90° knee flexion, damage to Kaplan fibres led to greater degrees of tibial internal rotation compared to when only the ALL was sectioned implicating that isolated ACL reconstruction might not restore the native knee kinematics efficiently at higher degrees of knee flexion [35, 36]. In a recent meta‐analysis, graft failure rates were higher in the isolated ACL reconstruction group when compared to the augmented ACLR group, there was a significant improvement in clinical outcomes with the addition of ALL reconstruction or lateral extra‐articular tenodesis (LET) to a revision ACL reconstruction surgery [8]. In another study, the return to sport rates were higher in patients who underwent revision ACL reconstruction with LET at 1‐year follow‐up as compared to those who underwent isolated ACLR [54].

## BIOMECHANICAL EFFECT OF LET/ALL

Multiple cadaveric studies have analysed the biomechanical effects of LET procedures, demonstrating that ACL reconstruction alone in the presence of anterior lateral injury results in residual internal rotation and laxity and anterior translation which significantly decreases when lateral augmentation procedures like LET or ALL reconstruction or combined with ACL reconstruction [19, 50, 52]. These procedures decrease the strain on the ACL graft in addition to increasing the rotatory knee stability. In a recent study the augmentation of ACLR by LET decreased the ACL graft force by 80%. A similar decrease in load on the graft was observed at 30° of knee flexion in a simulated setup [65]. Similar findings were seen in another study showing that the modified Lemaire technique reduced ACL graft forces by 61% [66]. This showed that LET could be considered for use in high‐risk patients due to its protective effect on ACL grafts.

A cadaveric study examined graft fixation at different knee flexion angles and found that the LET procedure restored the kinematics of the knee when the graft was fixed at 0°, 30° and 60° whereas ALL reconstruction was only successful when the graft was fixed keeping the knee in full extension [51].

There is a concern that LET might restrict internal tibial rotation and anterior‐posterior tibial translation. A recent study showed that both lateral augmentation (LET and ALLR) techniques decreased internal table rotation as the knee flexion angle increased [52]. Another study done on cadaveric specimens found that the centre of contact stress in the lateral compartment of the knee was shifted anteriorly when ACL reconstruction was combined with LET [64]. This also increased both the mean and peak lateral compartment contact stresses. In another study, it was found that femoral tunnel fixation corrected anterior tibial translation and rotational laxity more effectively than femoral cortical fixation in cadaveric models [99]. Various authors have also studied the biomechanical effects of LET using gait analysis and had shown restoration to pre‐injury gait patterns when ACL reconstruction was augmented with LET [13, 21]. In a recent study, it was found that at six months of follow‐up, LET resulted in a larger lateral compartment cartilage contact centre, which became comparable to the medial side at 12 months of follow‐up showing no significant difference [72].

## INDICATIONS AND CONTRAINDICATIONS OF LATERAL EXTRAARTICULAR PROCEDURES (LEAP)

LEAP can be added to primary or revision ACL in patients with one or more of the following indications [32, 33, 37, 38, 40, 58, 62, 70, 78, 80] (Table 1).

**Table 1:** Current indications and contraindications of LET procedures.

Indications of LET	Contraindications of LET
Younger individuals less than 25 years of age High‐grade, anterolateral lateral rotatory laxity (grade 2 or more pivot shift) Generalised ligament laxity (Beighton score>4) Knee hyperextension >10° Lateral coronal plane laxity Increased posterior tibial slope Concomitant lateral meniscal deficiency MRI evidence of anterolateral capsular injury Revision ACL reconstruction With high risk factors (any of the above)	Concomitant posterolateral corner injury/laxity Skeletal immature individual (risk of physeal damage) Pre‐existing lateral compartment knee osteoarthritis

Abbreviations: ACL, anterolateral ligament; LET, lateral extra‐articular tenodesis; MRI, magnetic resonance imaging.

## ALC INJURIES: OPERATIVE MANAGEMENT

Lateral extra‐articular procedures (LEAP) can be performed in patients with ACL‐deficient knees but the indications for this are not clearly defined. In patients having a markedly positive pivot shift test an extra‐articular procedure was effective at reducing the pivot shift [46]. The presence of large amounts of anterior translation in the lateral knee compartment might be an indication for extra‐articular procedures which may provide additional stability to the injured knee. After an ACL injury with associated ALRI, a combined extra and intra‐articular reconstruction is advocated to restore normal knee as kinematics. The extra‐articular reconstruction provides a more extended lever arm on the lateral side that limits tibial internal rotation effectively [25]. The extra‐articular tenodesis remains intact even if the intra‐articular graft fails, acting as a backup [23]. This extra‐articular tenodesis leads to decreased stress on the intra‐articular graft by up to 40%, showing its possible load‐sharing effect [23, 25, 27].

The effects of extra‐articular tenodesis on anterolateral rotatory knee stability have been well‐established with multiple navigation‐based studies. In a recent study, in vivo, knee kinematics were analysed before and after lateral tenodesis and ACL reconstruction. It was observed that during the Lachmann test, those patients who underwent isolated single bundle ACL reconstruction had a more anterior translation of the tibia as compared to those who underwent ACLR with lateral tenodesis [7]. Single‐bundle ACLR with lateral tenodesis works at par with the double‐bundle ACL reconstruction technique in reducing tibial internal rotation [69]. In contrast, the patients with the combined ACLR and extra‐articular reconstruction show better rotational control and lesser anterior displacement of the lateral compartment when performing an anterior drawer test. It has also been demonstrated that different extra‐articular tenodesis techniques have proven vital for better control of the tibial internal rotation and pivot shift phenomenon [3].

There are concerns regarding the development of early osteoarthritis in patients undergoing LEAP procedures, and the reason may be possible over‐tightening of the lateral compartment of the knee, which may lead to increased contact forces in the lateral compartment. On the contrary, without LEAP in patients with anterolateral rotational instability, there is inadequate control of tibial rotation and a higher risk of secondary meniscal and chondral injuries, this is also evident from the fact that certain authors have reported a graft failure rate of as high as 22% in ACLR without LEAP and increased risk of development of lateral compartment OA [67]. Certain studies have demonstrated no correlation between ALL reconstruction and early development of osteoarthritis compared to isolated ACL reconstruction [30, 63]. Similar findings have also been confirmed by a multicentric study conducted on 675 patients in which the authors observed that ALL reconstruction was not associated with arthritic degeneration of the knee at 12 years of follow‐up, which was instead seen mainly in patients who had undergone a medial meniscectomy [9]. Few studies have demonstrated significant improvement in high‐grade pivot shift and significant improvement in subjective scores like IKDC and Lysholm score and objective measurements like KT‐1000 with combined ACLR and ALL reconstruction [41].

In recent years, there has been an increased understanding of ALC amongst clinicians, which is evident by the increased interest of surgeons in performing the combined LEAP and the ACLR to restore knee kinematics and improve the long‐term outcomes of these patients. Pairing LET or ALL with ACLR could be especially advantageous for young patients at high risk of re‐injury, as it enhances stability and lowers the likelihood of requiring revision surgery due to graft failure. These procedures could be considered for high‐risk patients, such as those who are young and active in sports that involve pivoting, those demonstrating a high‐grade pivot shift, and those with generalised ligamentous laxity or knee hyperextension. Additionally, the presence of an associated Segond fracture, a chronic ACL injury, a deep lateral femoral notch sign, the presence of lateral coronal plane instability, an increased posterior tibial slope, a concurrent meniscus repair, or an ALC injury identified on MRI could be considered for an additional lateral extra‐articular procedure.

A variety of techniques that have been examined in various studies have been described in Table 2.

**Table 2:** Type of anterolateral extra‐articular procedure.

S. No.	Type of anterolateral extra‐articular procedure	Description
1)	ALLR	Free gracilis graft is passed under the ITB and over the lateral collateral ligament (LCL) [11]. The graft was passed into closed‐socket tunnels and fixed with interference screws. The tibial tunnel was located equidistant from the centre of Gerdy's tubercle (GT) and the anterior margin of the fibular head and 10 mm distal to the joint line [55, 71]. The femoral socket was located 5 mm proximal and posterior to the LCL's femoral insertion.
2)	Modified Ellison procedure	A 15 mm wide central strip of ITB detached distally by performing a thin GT osteotomy. This strip was passed deep to the LCL and the bone was fixed back to the GT bed. Fixation was with 3.5 mm suture anchors reinforced with a staple. The ITB defect was then closed.
3)	Modified deep Lemaire procedure	A 15 × 100 mm central strip of the ITB is passed under the LCL and fixed in the same tunnel as the ALLR with an interference screw.
4)	Modified superficial Lemaire	Performed using the same graft and the same femoral fixation site as modified deep Lemaire, but with the graft positioned over the LCL
5)	Modified MacIntosh	A 15 × 150 mm central strip of ITB is taken, passed underneath the LCL and through a further closed socket tunnel 70 mm proximal to the femoral epicondyle at the insertion of the lateral intermuscular septum, and fixed with an interference screw [20].

Abbreviation: ALLR, anterolateral ligament reconstruction.

Heterogenicity in LEAP is responsible for failing to yield valid data and results for each lateral extra‐articular reconstruction procedure. A few studies conducted recently have aided in ascertaining the efficacy and safety of some techniques [88, 90]. However, there needs to be more scientifically sound and appropriately drafted randomised controlled trials and level one studies to analyse the superiority of one type of LET or ALL reconstruction technique over the others.

As per the recent literature, the use of LEAPs has been increasing in both primary and revision ACLR over the past few years and lateral augmentation is being used more frequently with time. Surgical techniques have not changed much, with most surgeons preferring ITB autograft attached to Gerdy's tubercle, taken under the lateral collateral ligament (LCL), and anchored at a site proximal/posterior to the lateral femoral epicondyle [47].

## CLINICAL OUTCOMES

Recent evidence about clinical outcomes were summarised in Table 3. A meta‐analysis showed that the addition of LET to ACL reconstruction significantly reduced the rotational knee laxity as measured by the pivot shift test [46] but no difference in KT 1000/2000 measured anterior translation and patient‐reported clinical outcomes. These findings were confirmed by subsequent systematic reviews which reported significant improvement in pivot shift test but no significant improvement in patient outcome scores [1, 86]. In another study at 2 years follow‐up, there was a significant improvement in patient‐reported outcomes when LET was combined with ACL reconstruction [82]. Multiple authors have studied patient‐reported outcomes in patients with revision ACL reconstruction and lateral extra‐articular procedure and they found good results [4, 26, 94]. Combining LET with ACL decreases the failure rates and enhances the post‐operative functional scores [4]. A high return to sport rate and improved subjective outcomes were also shown in a study at a 5‐year follow‐up in a high‐risk cohort undergoing revision ACL reconstruction [94]. On the contrary, another study showed no difference in graft failure rates with or without LET in revision ACLRs in patients with preoperative anterior laxity less than 5 mm [26].

**Table 3:** Clinical outcomes.

Authors and Year	Type of study	Procedure	Primary or revision ACL	Clinical results	Biomechanical data	Failure rate	OA at follow up	Note
Pernin et al. 2010 [77]	Case series (*n* = 100) minimum 21 years of follow‐up	ACLR + LET	Primary	IKDC subjective score at final follow‐up was 74.7 6 18.6 points.	/	19.6% of patients	27% of patients with severe OA	1) Patients enroled between 1978–1983 2) Open arthrotomy 3) bone‐tendon‐bone graft and extra‐articular tenodesis (modified procedure of Lemaire and Combelles)
Hewison et al. 2015 [46]	Systematic review	Isolated ACLR/ACLR + LET	Primary	No difference of IKDC between LET group and isolated ACLR	Reduce pivot shift in ACL + LET group. No difference of KT‐1000 between isolated ACLR and ACLR + LET	/	/	/
Song et al. 2016 [86]	Systematic review	Isolated ACLR/ACLR + LET	Primary	No differences in objective IKDC scores and anterior knee stability	Significant reduction of residual pivot shift in ACLR + LET compared to isolated ACLR	/	/	/
Zaffagnini et al. 2017 [100]	Retrospective study (*N* = 29) Minimum 20 years of follow‐up	ACLR + LET	Primary	Mean Lysholm score was 85.7 ± 14.6; objective IKDC score, good or excellent in 86% of patients	(12% of patients) had >5‐mm manual maximum KT‐2000 side‐to‐side difference KiRA system documented positive pivot‐shift ( > 0.9‐m/s^2^ tibial acceleration side‐to‐side difference) in these 3 of 26 patients (12%).	One patient (2%)	Absence	Over the top technique + ALL reconstruction
Rowan FE et al. 2019 [82]	Retrospective study (prospensity score matching analysis)	Isolated ACLR/ACLR + LET	Primary	Post‐operative Lysholm score Tegner activity index and time to return to sport better in ACLR + LET group (*p* < 0.05)	/	No differences between groups	/	1) Minimum 2 years of follow up 2) An anatomical single bundle doubled gracilis and semitendinosus hamstring graft ( ≥ 8 mm) was used in all cases. Lemaire as LET
ALC consensus group et al. 2020 [2]	Systematic review	Revision ACLR + LET	Revision	Lysholm, IKDC, and KOOS scores indicated satisfactory results	KT‐1000 reported side‐to‐side difference > 5 mm in 17 patients (10%). 7 patients (1%) had Grade III of pivot shift	24 Failures (3.6%)	/	12 Studies included. 658 Patients included.
Alm et al. 2020 [4]	Retrospective study (*n* = 75 total)	Revision ACLR + LET/isolated revision ACLR	Revision	With the exception of post‐operative pain (VAS and KOOS) and the KOOS quality of life score, the group with combined revision ACLR and LET demonstrated significantly better post‐operative functional scores; the results were excellent compared to patients with isolated revision ACLR.	isolated ACLR showed a significantly increased incidence of a positive pivot‐shift test compared to ACLR + LET (*p* = 0.012). The post‐operative side‐to‐side difference measured via Rolimeter was also significantly elevated in the group with isolated revision ACLR (*p* = 0.001).	LET lead to significant decreased failure rates (5% vs. 21%) *p* = 0.045	/	/
Getgood et al. 2020 [38]	Randomised controlled trial (*n* = 618 patients total)	Isolated ACLR/ACLR + LET	Primary	No differences in terms of KOOS and pain	/	4% ACLR + LET vs. 11% isolated ACLR	7	Single‐bundle, hamstring tendon ACLR with or without LET performed using a strip of iliotibial band. 2 years of follow up
Eggelin et al. 2022 [26]	Retrospective cohort study (*n* = 78 total)	Isolated revision ACLR/revision ACLR + LET	Revision	No differences in terms of Lysholm, Tegner, IKDC, VAS	/	11% Ffailure ACLR + LET vs 13% failure isolated revision ACLR	/	Mean follow‐up of 28.7 ± 8.8 (24–67) months. LET (modified Lemaire)
Firth et al. 2022 [32]	Randomised controlled trial	Isolated ACLR/ACLR + LET	Primary	/	The addition of a LET and larger graft diameter were significantly associated with reduced odds of asymmetric pivot shift.	/	/	Graft: Autologous hamstring
Agarwal et al. 2022 [1]	Systematic review	Isolated ACLR/ACLR + LET/ACLR + ALLR	Primary	No clinical differences between groups	The addition of AEAPs to primary ACLR appears to result in improved rates of rotatory stability when comparing pivot shift test results	LET and ALL appear to result in reduced rates of graft failure, compared to isolated ACLR	/	*N* = 24 studies included
Viglietta et al. 2022 [98]	Cohort study (*n* = 165 total)	Isolated ACLR/ACLR + LET	Primary	No statistically significant differences in subjective scores between the two groups.	A side‐to‐side difference >5 mm on the KT‐1000 arthrometer evaluation was found in 9% of patients of ACLR and in 1% of patients in the ACLR + LET group (*p* = 0.01).	10% Failure isolated ACLR vs. 1% failure ACLR + LET	Patients underwent to isolated ACLR had a significantly higher OA grades than those in the ACLR + LET group for the overall tibiofemoral joint and the lateral compartment of the knee.	The mean follow‐up was 15.7 years.

Abbreviations: ACL, anterior cruciate ligament; ACLR, anterior cruciate ligament reconstruction; ALC, antero‐lateral corner; ALL, anterolateral ligament; ALLR, anterolateral ligament reconstruction; KOOS, Knee Injury and Osteoarthritis Outcome Score; IKDC, International Knee Documentation Committee; LET, lateral extra‐articular tenodesis; OA, osteoarthritis.

The STABILITY 1 trial has compared ACL reconstruction using hamstring autograft with and without LET including patients aged 14–25 years, having two of the following: a desire to return to high‐risk sports, Grade 2 or higher pivot shift, Generalised ligamentous laxity, knee recurvatum exceeding 10°. It was found that the combination of LET and ACLR significantly reduced rotatory instability and graft rupture at two years follow‐up [38]. A recent study utilised STABILITY I trial data and logistic regression analysis and showed that LET was protective for ACL graft while increased posterior tibial slope, early return to sports, younger age and high‐grade knee laxity increased the odds of graft rupture [32].

Even with overall positive patient outcomes, increased lateral compartment pressure observed in biomechanical studies is of great concern due to the anticipated risk of development of osteoarthritis in the lateral compartment of the knee. However, there is insufficient evidence to suggest an increased rate of OA with the combination of LET and ACLR. Case series consisting of 20–25 years of follow‐up show subjective patient satisfaction rates and no association of osteoarthritis development with LET [77, 100]. In a recent study, a significantly higher risk of long‐term OA was noted in patients with isolated ACL reconstruction compared to ACLR with LET at 15 years of follow‐up. Additionally, those undergoing combined ACLR and LET had lower graft rupture rates and better knee stability [98].

## ALL: CURRENT DEBATE AND FUTURE DIRECTIONS

There is current evidence that favours the use of lateral augmentation procedures with ACLR based on one of the best clinical evidence in the form of stability‐I trial, but it is conducted in patients where hamstring autograft is used for ACLR, the outcomes of LET augmentation when other commonly used grafts such as BPBT and quadricep autografts are used are yet to be unmasked by STABILITY‐II trial. This may further aid in our understanding of the long‐term outcomes of a supportive procedure that has a graft‐protective effect and has improved PROMs.

## AUTHOR CONTRIBUTIONS

Conceptualization: Amit Meena, Manish Attri, Luca Farinelli, Vicente Campos, Karan Rajpal, Shahbaz Malik, Darren de SA, Sachin Tapasvi and Christian Fink. Writing—original draft preparation: Amit Meena, Manish Attri, Luca Farinelli, Riccardo D'Ambrosi, Vicente Campos, Karan Rajpal, Shahbaz Malik, and Darren de SA. Writing—review and editing: Amit Meena, Manish Attri, Luca Farinelli, Riccardo D'Ambrosi, Vicente Campos, Karan Rajpal, Shahbaz Malik, Darren de SA, Sachin Tapasvi and Christian Fink. Supervision: Amit Meena, Sachin Tapasvi and Christian Fink. All authors interpreted the data, critically reviewed the work, made important contributions to the manuscript with their suggestions for improvement, approved the published version and agreed to be responsible for all aspects of the work. All authors have read and agreed to the published version of the manuscript.

## CONFLICT OF INTEREST STATEMENT

The authors declare no conflicts of interest.

## ETHICS STATEMENT

None declared.

## Data Availability

The data sets generated and analysed during the current study are available from the corresponding author on reasonable request.
